# Comparative Retrospective Cohort Study of Carotid-Subclavian Bypass versus In Situ Fenestration for Left Subclavian Artery Revascularization during Zone 2 Thoracic Endovascular Aortic Repair: A Single-Center Experience

**DOI:** 10.3390/jcm13175043

**Published:** 2024-08-26

**Authors:** Evren Ozcinar, Nur Dikmen, Cagdas Baran, Onur Buyukcakir, Melisa Kandemir, Levent Yazicioglu

**Affiliations:** Cardiovascular Surgery Department, Heart Center, Cebeci Hospitals, Ankara University School of Medicine, Mamak, Ankara 06340, Turkey; evrenozcinar@gmail.com (E.O.); cagdasbaran@gmail.com (C.B.); buyukcakironur@gmail.com (O.B.); melisakandemir1999@gmail.com (M.K.); leventyazicioglu@hotmail.com (L.Y.)

**Keywords:** in situ fenestration, carotid-subclavian bypass, zone 2 TEVAR, LSA revascularization

## Abstract

**Background:** Thoracic endovascular aortic repair (TEVAR) has become the first-line therapy for descending aortic disease. Recent studies have demonstrated that preventive revascularization of the left subclavian artery (LSA) in zone 2 TEVAR cases reduces the risk of neurological complications. However, there is no uniform consensus on the choice of revascularization techniques. Although carotid-subclavian bypass is considered the gold standard method, in situ fenestration techniques have also shown encouraging results. This study aims to compare the carotid-LSA bypass with in situ fenestration (ISF) for LSA revascularization and to discuss our treatment approach. **Methods:** We conducted a retrospective review of all patients undergoing zone 2 TEVAR with in situ fenestration (ISF) or carotid-subclavian artery bypasses for LSA revascularization at our institution between February 2011 and February 2024. Preoperative patient characteristics and primary outcomes, such as operative mortality, transient ischemic attack, stroke, and spinal cord ischemia, were analyzed between the groups. **Results:** During the 13-year study period, 185 patients underwent TEVAR procedures. Of these, 51 patients had LSA revascularization with zone 2 TEVAR; 32 patients underwent carotid-subclavian artery bypasses, and 19 underwent in situ fenestration. The technical success rate was 100%. Statistically, there was no significant difference between the groups in terms of primary outcomes such as stroke, transient ischemic attack, spinal cord ischemia, and death (*p* > 0.05). **Conclusions:** In situ fenestration (ISF) may be an effective and feasible method for LSA revascularization. With precise patient selection and in experienced hands, ISF appears to be associated with similar perioperative outcomes and mortality rates to the carotid-subclavian bypass.

## 1. Introduction

Historically, open surgery was the sole effective treatment for aortic arch pathologies, a complex class of aortic diseases [1]. However, since its advent, thoracic endovascular repair (TEVAR) has emerged as the preferred modality for managing many descending aortic pathologies, demonstrating outcomes that are equivalent to or superior to those of open surgical repair [1,2]. The proximal landing zone is crucial in endovascular therapy, along with the proper selection and sizing of the stent graft [3]. When an aortic lesion is situated just distal to the LSA or involving the LSA, covering the LSA orifice may be required to secure an adequate landing zone. The literature reports indicate that LSA coverage occurs in about 50% to 84% of patients undergoing TEVAR in acute cases [4,5,6]. The optimal approach for managing aortic pathologies involving the left subclavian artery (LSA)—designated as zone 2 by Ishimaru—remains a subject of debate [3,4]. The management of the left subclavian artery (LSA) during the repair of distal aortic arch disease presents unique anatomical and technical challenges, making it a procedure with a high risk of neurological complications and morbidity [5,6]. Recent studies have shown that the preventive of the LSA in zone 2 TEVAR cases significantly reduces the risk of anterior/posterior circulation stroke, as well as upper limb and spinal cord ischemia [5,6,7,8]. Despite the lack of consensus on the necessity for revascularization, numerous reports indicate high rates of neurovascular morbidity when revascularization is omitted [5,6]. Historically, revascularization options included the carotid-subclavian bypass or transposition. On the other hand, carotid-subclavian bypass is the gold standard revascularization procedure. Nevertheless, recent advancements in endovascular techniques have introduced alternative solutions such as chimney grafts, on-the-table fenestration, fenestrated and branched stent grafts, and in situ fenestration techniques. These innovations challenge the traditional carotid-LSA bypass or transposition methods [9,10,11]. Among these, in situ fenestration techniques include in situ needle fenestration (ISNF) and energy-based (radiofrequency or laser) in situ fenestration. These procedures involve performing in situ fenestrations during TEVAR, followed by the deployment of bridge stents aimed at preserving branch vessel patency. In situ fenestration (ISNF) of endovascular graft, though not a new concept, offers several appealing benefits. It allows for anatomical anterograde reconstruction, closely resembling open surgical repair. The technique is intuitive, relying on straightforward procedural steps, and it is highly adaptable, making it suitable for urgent situations [7]. In this study, we aim to compare carotid-LSA bypass with in situ needle fenestration (ISNF) for LSA revascularization during zone 2 TEVAR and to analyze the outcomes.

## 2. Materials and Methods

### 2.1. Study Design

**Figure 1:**
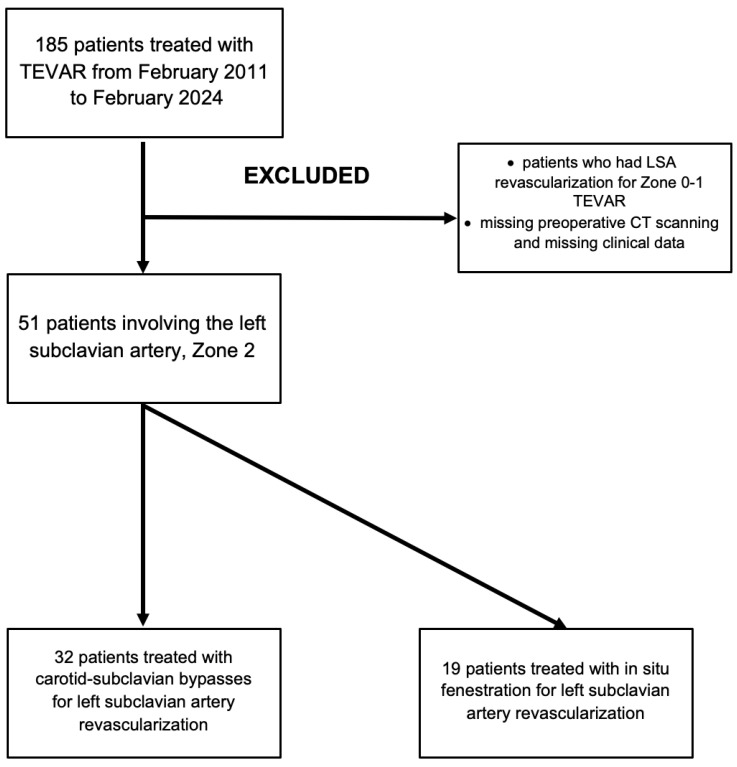
Flow diagram illustrating patient selection, exclusion, and treatment strategies. LSA: left subclavian artery; TEVAR: thoracic endovascular aortic repair; CT: computed tomography.

This study is a retrospective, physician-initiated cohort analysis conducted at a single center. We conducted a retrospective review of all patients who underwent zone 2 TEVAR for thoracic aortic pathologies—including aortic dissections, aneurysms, penetrating aortic ulcers, and blunt traumas—at our institution from February 2011 to February 2024. The study was conducted according to the guidelines of the Declaration of Helsinki, and the research ethics board at Ankara University approved this study (date: 17 May 2024, no:2024/340). Written informed consent was obtained from the patients. Clinical data were gathered in a dedicated, anonymized institutional health care database and analyzed retrospectively. The collected information included patient demographics, co-morbidities, the morphologic characteristics of the aortic lesion, the type of intervention, and the thoracic graft used, as well as postoperative outcomes such as complications, mortality, and the need for intervention during both the hospital stay and follow-up period. For patients undergoing multiple TEVAR procedures, only the first zone 2 case was analyzed. Exclusion criteria included patients with more than 2 cm of the aorta suitable for sufficient sealing, undergoing LSA revascularization for zone 0–1 TEVAR, and those missing preoperative computed tomography (CT) scans or clinical data (Figure 1). Primary outcomes such as operative mortality, transient ischemic attack, stroke, and spinal cord ischemia were analyzed between groups. 

The patients’ history and medical examination reports were thoroughly reviewed along with cardiac and pulmonary functions with a multi-step preoperative assessment. All informative intraoperative decisions concerning surgical technique and patient management were assessed. The patients’ computed tomography (CT) angiography scans were reviewed to define the extensive pathology, size, and the branches of the aortic arch.

All computed tomography angiographies (CTA) were performed using a spectral CT. The datasets from these CTAs were transported to a dedicated workstation, equipped with a specialized software for aortic and vascular reconstructions and calculations. The feasibility of TEVAR was assessed by the consensus among the physicians experienced in cardiovascular surgery. Access feasibility was determined by measuring both the inner and outer diameters of the largest iliac and femoral axis, with the narrowest point of the larger iliac-femoral axis selected as the minimum diameter. Aortic feasibility was evaluated based on the diameters, lengths, and angles of the aortic neck at the level of the supra-aortic trunks. The diameters were measured from the outer layer at the intended proximal landing zone (“zone 2”). The lengths were calculated as the stretched lengths along the inner, centerline, and outer curvatures between the distal edge of the left common carotid artery and the proximal edge of the LSA or aortic neck. The distance between the LSA and the ipsilateral vertebral artery take-off was measured as the stretched centerline length from the LSA take-off to the origin of the vertebral artery. The feasibility of the left subclavian artery vessel was determined by evaluating its diameter, length to the level of the ipsilateral artery, and angulation from the aortic arch. Angulations were assessed using the multiplanar projection of the arch. 

A patient was placed in a supine position, and under general anesthesia, a 14-G needle is inserted into the lumbar interspaces, L3–L4 or L4–L5, and the cerebrospinal fluid (CSF) catheter was advanced until the CSF was obtained. The CSF pressure was monitored during the operation period for achieving the goal target <15 mmHg. We inserted a rapid pacing diode into the right ventricle via the right internal jugular vein for the quick maneuver of the thoracic graft. We placed a transesophageal echocardiogram (TEE) for ensuring the precise deployment of the stent graft. To the patient’s forehead position, diodes for near infrared spectroscopy INVOS 5100C (Somanetics, Troy, MI, USA) were attached bilaterally for screening regional oxygen saturation. All procedures were performed under general anesthesia in a hybrid operating room. Prophylactic lumbar cerebrospinal fluid pressure monitoring and drainage were implemented. Using the rapid pacing procedure, draining cerebrospinal pressure, and monitoring, along with maintaining the patient in a hypertensive state during the intervention, were key strategies aimed at preventing the adverse events associated with the procedure (e.g. Spinal ischemia, stroke, etc.).

The selection of the thoracic stent graft was left to the surgeons’ discretion, with options including Ankura (Lifetech, Shenzhen, China) and the Valiant (Medtronic, Minnepolis, MN, USA). Typically, the thoracic stent graft was oversized by approximately 0–10% for aortic dissections, blunt traumas, and intramural hematomas, and by 10–20% for aneurysms and penetrating ulcers. 

Postoperative care involved the best medical treatments, including antiplatelet and antihypertensive drugs. The ISNF group specifically received dual antiplatelet therapy. Follow-up evaluations occurred at 3, 6, 12, 18, and 24 months post-operation via CT imaging to assess graft patency and identify endoleaks. Over 90% of patients completed the 24-month follow-up. Baseline characteristics of the cohort are detailed in Table 1.

### 2.2. Carotid-LSA Bypass Technique

The carotid-LSA bypass and subsequent TEVAR were performed in separate sessions. For the open surgical procedure, the first step involved performing a carotid-LSA bypass, followed by a TEVAR intervention approximately 2 h later. After administering general anesthesia, the patient was positioned supine, with the neck extended and the head tilted to the contralateral side. Standard cardiovascular monitoring (ECG, pulse oximetry, invasive arterial blood pressure, and cerebral oxygen saturation) was used. A 5 cm supraclavicular incision, beginning medially over the clavicular head of the sternocleidomastoid (SCM) muscle, parallel to the left clavicle, was made. Then, the platysma is incised. Care was taken to avoid phrenic nerve injuries while mobilizing the pre-scalene fat pad and dissecting beneath the anterior scalene muscle. The LSA and left common carotid artery (LCCA) were surgically exposed. After 5000 units of intravenous heparin administration, a Dacron 8 mm graft was anastomosed end-to-site to the LSA at the origin of the left vertebral artery, tunneled beneath the internal jugular vein and anastomosed to the LCCA with an 8-mm Dacron graft, and a 6–0 polypropylene suture was run. Once the graft was prepared, it was carefully deaired and trimmed to the appropriate length, usually requiring only 5 cm. The left subclavian artery (LSCA) is then clamped and opened, allowing the distal anastomosis to be performed using a similar technique as before. The bypass procedure can typically be completed in 45 min. Then, the proximal portion of the LSA was either ligated or coiled just before the orifice of the vertebral artery. Following this, a TEVAR procedure was carried out in the second session. 

### 2.3. In Situ Fenestration

**Figure 2:**
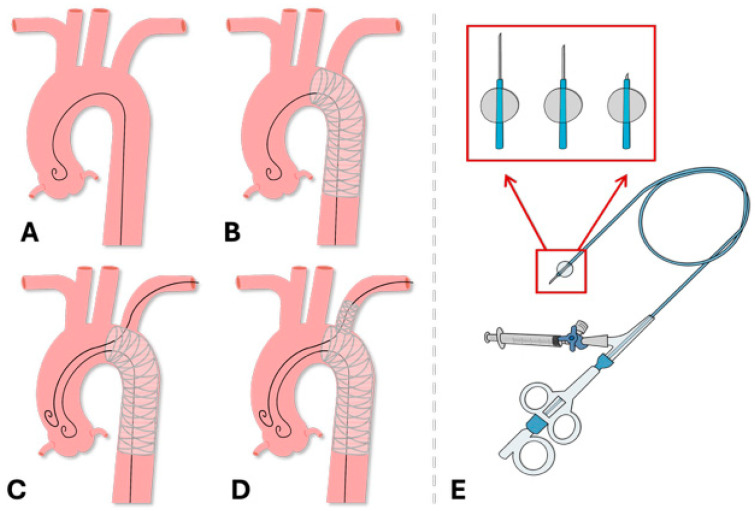
Operational details of left common carotid artery In Situ Needle Fenestration. (**A**) The guidewire was placed in the ascending aorta; (**B**) the aortic stent graft was deployed in the aorta; (**C**) the guidewire was advanced retrogradely through the brachial artery into the aorta; (**D**) fenestration of the left subclavian artery was performed, and the bridging covered stent was placed; (**E**) the in situ fenestration puncture system was utilized.

All patients were evaluated for endovascular repair suitability using preoperative CT angiography with exclusion criteria for thoracic endografts including a landing zone length of less than 20 mm, a landing zone diameter exceeding 40 mm, or the involvement of all cervical branches in the aneurysmal wall (zone 0) landing cases. Based on preoperative three-dimensional CT imaging, the Ankura thoracic stent graft was used for all 19 patients undergoing ISNF, owing to the presumed advantages of its expanded-polytetrafluoroethylene (e-PTFE) membrane, which is believed to facilitate easier puncturing and fabric durability. The operation details are shown in Figure 2.

**Figure 3:**
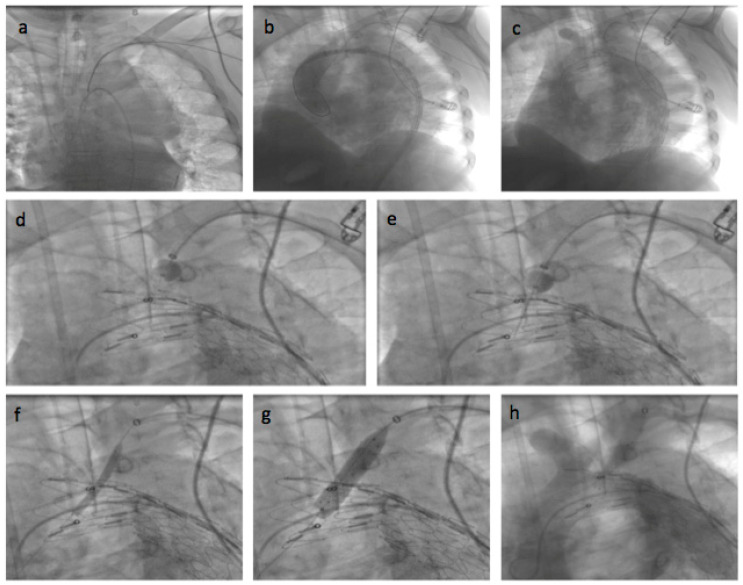
Angiographic images of left common carotid artery in situ needle fenestration: (**a**) Guidewire inserted into the ascending aorta; (**b**) aortic stent graft was advanced in the descending aorta in the unopened state, and the guidewire was retrogradely inserted into the aorta via the brachial artery; (**c**) the aortic stent graft was placed in the aorta; (**d**) the in situ needle fenestration puncture system was advanced to the left subclavian artery ostium; (**e**) the stent graft was punctured with the needle; (**f**) the fenestration was expanded with a balloon; (**g**) the stent was implanted in the fenestration; (**h**) the operation was evaluated with control digital subtraction angiography.

**Figure 4:**
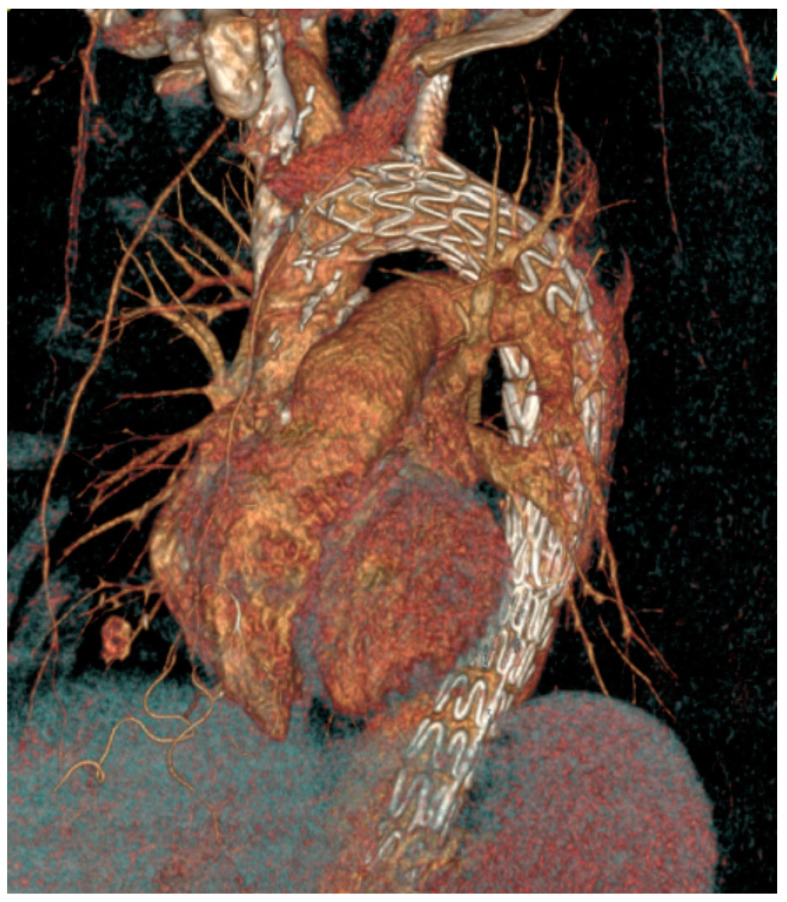
Postoperative computed tomography angiography (CTA) 3D image after the in situ needle fenestration thoracic endovascular aortic repair (TEVAR) procedure.

General anesthesia was administered. Vascular access was achieved through the common femoral artery via a cut-down. The FuThrough puncture system (Lifetech Scientific, Shenzhen, China) is designed with a self-centering balloon catheter and an adjustable 20-gauge needle. The puncture system for left subclavian artery (LSA) fenestrations was introduced via the left brachial artery, with an additional cut-down at this site. In situ needle fenestration (ISNF) was initiated following the deployment of the endograft. A steerable sheath (Fustar-Lifetech Scientific, Shenzhen, China) is inserted through the brachial access. The sheath is carefully positioned to be as perpendicular as possible to the greater curvature of the aortic arch, ideally making direct contact with the outer curvature of the thoracic endograft. To ensure stability, the balloon at the tip of the adjustable puncture needle is inflated, with the penetration depth pre-set on the device. The fenestration process involved passing a 0.018 or 0.035 guidewire through the puncture site (advanced into the ascending or descending aorta), which was then widened with a small-sized balloon (4–6 mm). Subsequently, the ISNF is connected with a balloon-expandable chrome cobalt ePTFE stent graft (BeGraft-Bentley, Hechingen, Germany), oversized by about 10%, and potentially antegrade up to 5 mm within the fenestration. The angiographic images during the procedure are shown in Figure 3, and Figure 4 shows the postoperative CT angiography 3D image. The Ankura stent graft was selected for all ISNF group cases due to its properties that facilitate easy puncture and dilatation. This choice was based on its ability to accommodate the technical requirements of ISNF effectively. (Figure 3 and Figure 4).

### 2.4. Statistical Analysis

Categorical variables were reported as frequencies, and continuous variables as means ± standard deviations or medians with interquartile ranges (IQR). The chi-square or Fisher’s exact tests were used for categorical data analysis, and the Mann-Whitney U-test for continuous variables. Mortality-free time related to the operation was analyzed using the Kaplan-Meier method, presenting means with standard deviations and hazard ratios with 95% confidence intervals. A *p*-value less than 0.05 was considered statistically significant. Analyses were performed using SPSS software (SPSS Inc., Chicago, IL, USA, version 20).

## 3. Results

During the 13-year study period, 185 patients underwent the TEVAR procedure. Among these, 51 patients had LSA revascularization with zone 2 TEVAR; 32 patients underwent carotid-subclavian artery bypasses, and 19 underwent in situ needle fenestration. The median age was 60.81 ± 13.25 years, with a demographic distribution of 11 (21.56%) females and 40 (78.44%) males. Forty-five patients underwent elective surgery, while six were operated on urgently. Diagnoses included thoracic aortic aneurysms in 29 patients (56.86%), type B dissections in 20 patients (39.22%), a penetrating aortic ulcer in one patient (1.96%), and a blunt trauma in one patient (1.96%). 

Fifteen patients were asymptomatic, while 36 presented with symptoms such as chest pain, back pain, and shortness of breath. The primary underlying risk factors were hypertension (73%) and a history of smoking (58.82%). Primary technical success was achieved in all cases (51/51, 100%). 

In the carotid-LSA bypass group, patients experienced complications related to surgical incision and vascular issues during follow-up. One patient (1/32, 3.12%) suffered from a lymphocele, two patients (2/32, 6.25%) had pseudoaneurysms, and one patient (1/32, 3.12%) had a hematoma. No access-related complications were observed in the ISNF group. 

The mean operation time was 138 min (range 64–248) in the carotid-LSA group and 78 min (range 52–124) in the ISF group. These time periods were measured from the beginning of the surgical incision to the last suture of skin, with the ISNF group having a significantly shorter median operation time.

**Figure 5:**
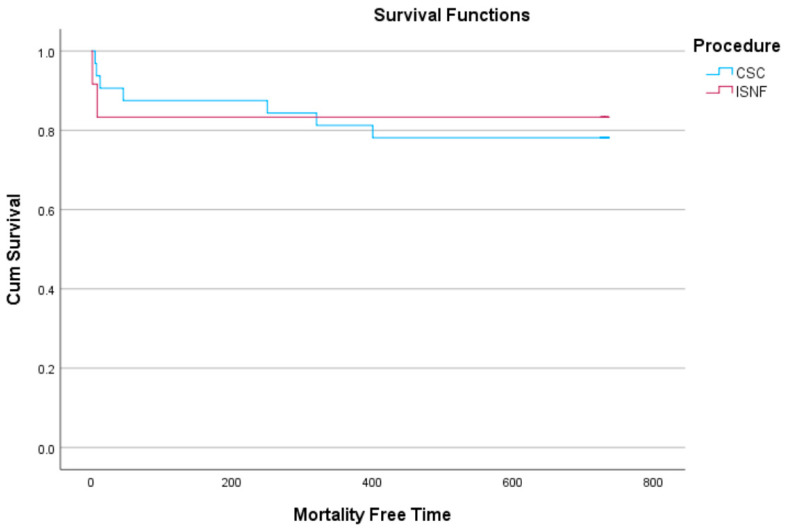
Kaplan Meier analysis for 2 years mortality between the groups (CSC: Carotid-Subclavian Bypass group; ISF: In situ Fenestration group).

Early mortality was 9.8% (n = 5) across all patients who underwent LSA revascularization. In the ISNF group, two patients (2/19, 10.5%; one patient due to renal insufficiency, one patient due to respiratory failure) died within the first 30 days post-operation, while three patients (3/52, 5.76%; two patients due to pulmonary dysfunction, and one patient due to cardiac failure) in the carotid-LSA bypass group died. During follow-up (1–24 months), four additional deaths (three patients due to malignancy, one patient due to respiratory failure) occurred in the carotid-LSA bypass group with no late-term mortality observed in the ISNF group. There was no significant difference in mortality between the carotid-LSA bypass and ISNF groups. The Kaplan-Meier analysis is shown in Figure 5.

All patients who underwent TEVAR were routinely transferred to the cardiovascular surgery intensive care unit postoperatively and then to the ward. The total hospital stay was 15.03 ± 25.75 days for the carotid-LSA group and 10.36 ± 10.27 days for the ISNF group. 

During the post-surgical follow-up, two patients in the carotid-LSA group experienced a stroke, and one patient had spinal cord ischemia despite prophylactic cerebrospinal fluid drainage catheter use. No transient ischemic attacks were observed. In contrast, in the ISNF group, one patient had a stroke, and one patient experienced a transient ischemic attack. No instances of spinal cord ischemia were observed in the ISNF group.

Three patients in the carotid-LSA group were diagnosed with type 1 endoleak during follow-up with CTA scans, with diagnosis times at 3, 10, and 12 months, respectively. No endoleaks were found in the ISNF group during follow-up. Additionally, two patients (due to type B false lumen reconstruction with the extension endograft procedure) in the ISNF group and three patients (one patient due to patency of bypass graft, one patient due to late pseudoaneurysm, one patient extension endograft procedure) in the carotid-LSA group required reintervention. Major adverse events were not observed in subsequent follow-ups. Statistically, there was no significant difference between the groups regarding primary outcomes such as stroke, transient ischemic attack, and spinal cord ischemia (*p* > 0.05). When the patients who completed 24 months of follow-up were reviewed, the patency rates were found to be 100% in the ISNF group, while it was detected to be 93.75% in the carotid-LSA bypass group. During the postoperative period there were no observed any cervical nerve complications in the carotid-LSA bypass and the ISNF group. The outcomes are also detailed in Table 2.

## 4. Discussion

Thoracic endovascular aortic repair (TEVAR) is a critical procedure for treating aortic aneurysms, dissections, penetrating aortic ulcers, and blunt traumas involving the descending aorta. TEVAR offers reduced mortality and morbidity rates compared to open surgery [1,2]. However, TEVAR for aortic arch aneurysms presents challenges due to anatomical restrictions and the presence of cervical branches. Coverage of the left subclavian artery (LSA) during TEVAR is necessary in 10% to 50% of patients to achieve an adequate proximal landing zone and graft seal [12,13,14]. The LSA is vital for left upper extremity circulation, spinal cord perfusion via left vertebral artery, and coronary circulation in patients with a left internal mammary artery bypass graft. Recent studies have demonstrated that preventive revascularization of the LSA in zone 2 TEVAR cases reduces the risk of anterior/posterior circulation stroke, as well as upper limb and spinal cord ischemia [5,6,7,8]. Traditionally, LSA revascularization has been performed surgically via transposition or more commonly by left carotid artery bypass [15]. However, recent advancements in endovascular techniques have introduced alternative solutions, such as chimneys, periscopes, surgeon-modified endografts, branched-fenestrated endografts, and in situ graft fenestration [9,11,16]. In situ fenestration (ISF), first described by McWilliams et al. in 2004, is one such technique [17]. Our study aimed to compare carotid-LSA bypass and ISNF for LSA revascularization.

The carotid-LSA bypass is recognized as a valuable technique for LSA revascularization [3,8,13]. Mortality rates reported in the literature for this procedure range from 1.4% to 5% [3,6,13]. However, our study observed a higher early mortality rate. Factors contributing to this include demographic characteristics, preoperative status, and comorbidities of the patients. Additionally, the small number of patients in our study likely influenced the high mortality rate. We routinely utilized a cerebrospinal fluid drainage catheter system and rapid pacing procedure before covering the thoracic aorta with stent grafts longer than 200 mm. Despite this precaution, spinal cord ischemia occurred in one patient in the carotid-LSA bypass group, aligning with the reported range of 0–10% in the literature [3,6,13]. 

Various fenestration techniques, including needle, radiofrequency, and laser, have been described. The success rates, complications, and technical difficulties associated with these techniques remain topics of discussion in the literature. Long-term durability is a primary concern, influenced by the choice of the fenestration technique and dilatation balloon and the fabric composition of the TEVAR stent graft [18,19].

The evidence is insufficient to definitively favor one retrograde in situ fenestration method over another. The laser fenestration technique, with an initial fenestration size of 2–3 mm, allows direct dilation with a 6 mm angioplasty balloon, avoiding the need for pre-dilation with smaller balloons as required by the needle technique [7,20]. Laser fenestrations do not produce visible emboli or clots [21]. However, some studies suggest that laser fenestration may weaken the TEVAR stent graft fabric more than ISNF [22]. Conversely, ISNF is suitable unless the LSA has an acute angle from the aorta [21].

When selecting a suitable aortic stent graft for in situ fenestration, the fenestration method and size are key considerations. Zenith grafts, made of Dacron, are stronger than Talent and Endurant stent grafts due to their multifilament structure [18,23]. Ankura stent grafts are preferred for their ease of puncture and dilation, producing larger, higher-quality elliptical fenestrations due to their ePTFE (expanded polytetrafluoroethylene) structure [24,25]. The choice of stent graft should be based on the fenestration technique and the stent graft’s properties and durability [18]. The primary controversy surrounding in situ fenestration lies in the uncertainty regarding the long-term durability of unreinforced fenestration. Without the reinforcement provided by a nitinol or stainless-steel ring, the durability of the fenestration depends solely on the composition of the graft fabric and the quality of the fenestration itself. The choice of an angioplasty balloon and bridging stent also plays a crucial role in the procedure. The size of the created fenestration is influenced by the fabric of the stent graft and the size and type of the angioplasty balloon used. Needle fenestration usually produces a smaller initial fenestration of about 1 mm, in contrast to laser fenestration typically creates a 2 to 3 mm initial fenestration, requiring pre-dilatation with a smaller balloon before further dilatation. When it comes to bridging stents, the most common used stent to connect the newly formed fenestration to the target artery is a 6 to 10 mm covered stent. Techniques for deploying these stents vary, while others employ a self-expanding or balloon-mounted covered stent, reinforced with a bare-metal stent. We always preferred covered stents in our cases. 

In situ fenestration has been most commonly used for revascularization of the left subclavian artery (LSA) following planned coverage with an endograft. Despite early successes, the widespread adoption of this technique is limited by the lack of long-term follow-up data, with concerns centered on the long-term durability of unreinforced fenestrations. Long-term studies are needed to fully assess the safety and efficacy of the technique. 

Patient selection is another critical consideration. In situ fenestration can be particularly challenging in patients with tortuous subclavian artery anatomy. The catheter must often assume a complex double-curved shape to achieve an appropriate angle for fenestration. Ensuring the fenestration angle is as close to 90 degrees as possible, centered within the stent graft, and not adjacent to supporting stents is essential for achieving a high-quality fenestration. 

The ISNF technique offers a less invasive alternative to the carotid-subclavian artery bypass, allowing for complete endovascular revascularization of the LSA post-TEVAR and resulting in quicker patient recovery [3]. In our study, the primary technical success rate was achieved in all cases (100%). The carotid-LSA bypass group experienced four access-related complications, while none were observed in the ISNF group. The ISNF group also had shorter operation times compared to open surgical LSA revascularization. The necessity for two separate operations in the carotid-LSA bypass group contributed to a slower recovery process. 

For LSA revascularization following TEVAR, the ISNF technique has some advantages other than fewer access-related complications, rapid recovery, and short operation time. In our study, we found 100% patency rates in the ISNF group at 24-month follow-up. However, the disadvantage is that the long-term results of this technique are not well known.

The literature reports mortality rates for the ISNF group ranging from 2% to 15%, consistent with our study’s findings [18,24,26]. Stroke rates were lower in ISNF group compared to the literature [18,27,28]. In our study, strict patient selection criteria based on CTA images resulted in no endoleaks during follow-up, and only two deaths within the first 30 days post-procedure, with no long-term mortality in the ISNF group.

Overall, our study’s findings align with the current literature, showing no significant difference between the groups in primary outcomes such as operative mortality, transient ischemic attack, stroke, and spinal cord ischemia (*p* > 0.05). There is no proven superiority of techniques over the other to date [1,3,6,12].

### Study Limitations

This study is limited by its retrospective nature and the single-center design, potentially affecting the generalizability of the results. The study may also be affected by sampling bias to the lack of comparison with alternative fenestration techniques, the choice between the carotid-subclavian bypass and ISNF, which was influenced by the patients’ overall condition and angio-anatomic criteria. Physician preferences and the small cohort size could lead to significant bias in the interpretation of the findings. The predominance of the men in the cohort reflects a common trend in TEVAR treatments but may limit the applicability of findings across different demographics. The anatomical feasibility was assessed using the current IFUs of the Ankura endograft, which may be subject to change in the future. These limitations could impact the generalizability of our findings. However, our data align well with the feasibility of TEVAR with ISF. Despite these limitations, this study offers valuable insights into the comparative effectiveness of the carotid subclavian bypass and ISNF, supported by a relatively large sample size and midterm follow-up results. To enhance the validity and generalizability of these findings, future research should focus on addressing these limitations through prospective designs, larger sample sizes, multi-center collaboration, and longer follow-up durations.

## 5. Conclusions

The emerging endovascular technique offer viable alternatives to traditional surgical methods like carotid-subclavian bypass or transposition for zone 2 TEVAR procedures. Among these, ISNF has shown itself to be a competitive option when compared to carotid-subclavian bypass. However, it is successful application requires careful patient selection and a high level of experience with the technique. Given the current limitations in long-term durability data, in situ fenestration is generally reserved for urgent cases. Surgeons should also be aware that in situ fenestration represents an off-label use of stent grafts. As an off-label approach, ISNF may affect the long-term durability of the stent graft. Further advancements in both equipment and techniques are necessary to enhance the reliability of ISNF. Despite these challenges, ISNF has proven to be effective and feasible for LSA revascularization, demonstrating similar perioperative outcomes and mortality rates as traditional methods. Our findings suggest that with careful patient selection and experienced execution, ISNF can be a potential strategy for managing complex aortic pathologies. Additionally, long-term durability data are essential before ISNF can be widely adopted.

## Figures and Tables

**Table 1:** Baseline, postoperative, and intraoperative patient characteristics.

	All Patients (n = 51)	ISF (n = 19)	Carotid–LSA Bypass (n = 32)	*p* Values
Gender (%)				
Male	40	16	24	
Female	11	3	8	0.405
Age, years				
Mean	60.81 (±13.25)	59.58 (±14.68)	61.29 (±12.63)	
Range	19–85	19–76	35–85	0.958
Timing of Treatment				
Elective	45	14	31	
Emergent	6	5	1	0.056
Main Diagnosis				
Aortic Aneurysm	29	13	16	
Aortic Dissection	20	5	15	
Penetrating Aortic Ulcer	1	0	1	
Blunt Trauma	1	1	0	0.212
Clinical Presentation				
Asymptomatic	15	8	7	
Symptomatic	36	11	25	0.259
Comorbidities				
Hypertension	37	10	27	0.227
Diabetes Mellitus	9	4	5	0.663
Chronic Heart Failure	6	2	4	0.658
Chronic Kidney Disease	10	4	6	0.687
Chronic Obstructive Pulmonary Disease	8	3	5	0.933
Coronary Artery Disease	18	4	14	0.733
Peripheral Vascular Disease	3	1	2	0.375
Smoking History	30	7	23	0.124
Prior Aortic Surgery	8	3	5	0.933
Stroke History	3	1	2	0.807

Abbreviations: ISF: In Situ Fenestration group; LSA: left subclavian artery.

**Table 2:** Outcomes.

Mean Operation Time(min)		78 (52–124)	138 (64–248)	0.034
0–30 Days Mortality	5	2	3	0.603
1–24 Months Mortality	4	0	4	0.556
Total Hospital Duration	13.86 ± 22.97	10.36 ± 10.27	15.03 ± 25.75	0.116
Pre-op Hemoglobin Level (g/dL)	11.66 ± 1.89	12.27 ± 2.36	11.42 ± 1.66	0.452
Post-op Hemoglobin Level (g/dL)	10.42 ± 1.79	10.66 ± 2.07	10.32 ± 1.70	0.351
Pre-op Creatinine Level (mg/dL)	1.02 ± 0.50	1.04 ± 0.33	1.01 ± 0.56	0.140
Post-op Creatinine Level (mg/dL)	1.09 ± 0.58	1.23 ± 0.50	1.03 ± 0.61	0.68
Major Adverse Events in 30 days				
Stroke	3	1	2	0.807
Transient Ischemic Attack	1	1	0	0.273
Spinal Cord Ischemia	1	0	1	0.536
Endoleaks				
Type 1	3	0	3	0.551
Type 2	0	0	0	*
Type 3	0	0	0	*
Type 4	0	0	0	*
Patency During 24 Months	49	19	30	0.624
Necessity of Reintervention	5	2	3	0.915

*: There is no difference since both groups are zero. Statistical significance cannot be calculated.

## Data Availability

The original contributions presented in the study are included in the article, further inquiries can be directed to the corresponding authors.
